# Comparative assessment of the effective population size and linkage disequilibrium of Karan Fries cattle revealed viable population dynamics

**DOI:** 10.5713/ab.23.0263

**Published:** 2023-11-02

**Authors:** Shivam Bhardwaj, Oshin Togla, Shabahat Mumtaz, Nistha Yadav, Jigyasha Tiwari, Lal Muansangi, Satish Kumar Illa, Yaser Mushtaq Wani, Sabyasachi Mukherjee, Anupama Mukherjee

**Affiliations:** 1 Animal Genetics and Breeding Division, ICAR- National Dairy Research Institute (NDRI), Karnal 132001, Haryana, India; 2 Department of Animal Genetics and Breeding, CVAS, RAJUVAS, Bikaner 334001, Rajasthan, India; 3 Livestock Research Station, Garividi Sri Venkateswara Veterinary University, Andhra Pradesh 535101, India

**Keywords:** Composite Cattle, Effective Population, Genomic Diversity, Karan Fries, Linkage Disequilibrium

## Abstract

**Objective:**

Karan Fries (KF), a high-producing composite cattle was developed through crossing indicine Tharparkar cows with taurine bulls (Holstein Friesian, Brown Swiss, and Jersey), to increase the milk yield across India. This composite cattle population must maintain sufficient genetic diversity for long-term development and breed improvement in the coming years. The level of linkage disequilibrium (LD) measures the influence of population genetic forces on the genomic structure and provides insights into the evolutionary history of populations, while the decay of LD is important in understanding the limits of genome-wide association studies for a population. Effective population size (N_e_) which is genomically based on LD accumulated over the course of previous generations, is a valuable tool for e valuation of the genetic diversity and level of inbreeding. The present study was undertaken to understand KF population dynamics through the estimation of N_e_ and LD for the long-term sustainability of these breeds.

**Methods:**

The present study included 96 KF samples genotyped using Illumina HDBovine array to estimate the effective population and examine the LD pattern. The genotype data were also obtained for other crossbreds (Santa Gertrudis, Brangus, and Beefmaster) and Holstein Friesian cattle for comparison purposes.

**Results:**

The average LD between single nucleotide polymorphisms (SNPs) was r^2^ = 0.13 in the present study. LD decay (r^2^ = 0.2) was observed at 40 kb inter-marker distance, indicating a panel with 62,765 SNPs was sufficient for genomic breeding value estimation in KF cattle. The pedigree-based N_e_ of KF was determined to be 78, while the N_e_ estimates obtained using LD-based methods were 52 (SNeP) and 219 (genetic optimization for N_e_ estimation), respectively.

**Conclusion:**

KF cattle have an N_e_ exceeding the FAO’s minimum recommended level of 50, which was desirable. The study also revealed significant population dynamics of KF cattle and increased our understanding of devising suitable breeding strategies for long-term sustainable development.

## INTRODUCTION

India is endowed with diverse cattle breeds, the majority of which exhibit low milk yields, prolonged calving intervals, and delayed age at first calving, typical for a tropical climate [1,2]. The planners thought that the most expedient way to enhance the productivity of these less-producing indigenous cattle was to breed them with exotic cattle that were well-known for their high milk production, early maturity, and reproductive efficiency. One such crossbreeding project was initiated at ICAR-NDRI, Karnal in 1971, which involved crossing indicine Tharparkar (T) cows with exotic Holstein Friesian (HF), Brown Swiss (BS), and Jersey (J) bulls [3].

The erstwhile Institute breed committee assessed the diverse levels of exotic inheritance in various cattle groups and suggested that stabilizing the population at 62.5% exotic level will be beneficial to maintain higher productivity. The outcome resulted in the development of a composite cattle known as Karan Fries (KF) since 1982. The breed has since been maintained at this level of exotic inheritance by selective breeding. Presently, the sixth generation of KF cattle has been maintained in the Livestock Research Complex of ICAR-National Dairy Research Institute, Karnal, India [4].

Various cattle populations, including crossbreds kept in organized herds, have experienced a decline in the level of genetic diversity due to increased selection intensity over time [5]. The decline in genetic variation poses a significant threat to the sustainability of these cattle breeds [6]. An earlier genealogical study utilizing pedigree data of KF cattle has shown few alarming results, with the individual cow having a high inbreeding coefficient of 31.25% in the herd [4]. This situation necessitated a thorough study for robust analysis of the population diversity in KF cattle using high-throughput genomic data. One of the critical genetic parameters of the population that reveal the evolutionary history and genetic diversity is the effective population size, N_e_ [7], which refers to the size of an idealized population undergoing the same rate of genetic drift as the population being studied [8]. The N_e_ has a dual role that aids our understanding of genetic dynamics in populations. Firstly, N_e_ provides insight into observed genetic variation and its distribution within a population, viewed from a retrospective perspective. By analysing the past, N_e_ helps explain how the patterns of genetic diversity have developed over time. Secondly, N_e_ offers a predictive perspective, particularly valuable when considering small breeding populations. It can estimate the potential loss of genetic variation in the future and shed light on the survival prospects of these populations. In essence, N_e_ serves as a valuable tool for delving into both the historical patterns of genetic diversity and the possible genetic trajectories that lie ahead for populations [9].

Effective population size (N_e_) also provides valuable insights into the potential for adaptation, genetic drift, and the risk of inbreeding in a population. The availability of genomic data has revolutionized the process of estimation of N_e_, as it can overcome the limitations of traditional pedigree-based methods which can be biased particularly in populations with complex breeding histories [10]. Furthermore, N_e_ can help to identify potential bottlenecks and genetic drift in composite cattle breeds, which can lead to the loss of beneficial alleles and an increased risk of inbreeding. By detecting these events, breeders can take measures to increase genetic diversity and minimize the risk of inbreeding depression in the population [11]. In addition, the information generated can help to adopt suitable breeding strategies to ensure the long-term sustainability of composite cattle breeds. Cumulative selection pressure over generations results in a reduction in N_e_ due to its impact on genetic drift [12]. If the estimated N_e_ is low, it suggests that the population may benefit from introducing new genetic material to increase genetic diversity [11].

The methods for N_e_ estimation can be broadly classified as pedigree-based, demographic, and marker-based approaches [13]. Pedigree-based and demographic approaches require extensive record keeping and do not permit judgments about the historical N_e_; therefore, the focus has now turned to the marker-based approach, with a preference for the linkage disequilibrium (LD) based technique, due to the abundance of genotype data and reducing cost of genotyping [14].

Linkage disequilibrium is another population parameter that refers to the non-random association of alleles at different loci in a population, which arise due to the non-random assortment of genes during meiosis [15]. Linkage disequilibrium has a significant role in population genetics. It helps us uncover the evolutionary past of populations and how much natural selection has influenced them. LD also reveals information about the population’s history, like changes in size, migrations, and mixing between different groups [15]. Additionally, LD can be used to detect the presence of functional variants, such as those associated with complex diseases [16]. The decay of LD over time is particularly relevant in the context of genome-wide association studies (GWAS), where the goal is to identify genetic variants associated with complex traits or diseases. The decay of LD over time can limit the power of GWAS, as the signal of association between a genetic variant and a trait may be lost due to the breakdown of LD between the variant and the causal variant [17]. Therefore, the degree of LD between markers is one valuable criterion to determine the minimum number of markers (marker density) required for conducting various genomic studies [18].

Under this background with the ongoing KF breed development programme for the last four decades, the present investigation was taken to estimate the N_e_ using genealogical and genomic tools, and to study the pattern of LD in KF cattle to get insight of the population dynamics. Genotype data of three other well-stabilized composite breeds (Santa Gertrudis [SG], Brangus [BR], and Beefmaster [BM]) and one of its parental breeds (HF) were utilized along with KF for comparative purpose.

## MATERIALS AND METHODS

### Animals genotyping and quality check

The present study was conducted on KF composite cattle maintained at Livestock Research Complex, ICAR-NDRI, Karnal, India. We also obtained genotype data of three other well-known composites from across the world, i.e. SG, BM, and BR. Purebred HF cattle genotype data were also used as a control population from Widde ( http://widde.toulouse.inra.fr/widde/) (Table 1).

KF animals (n = 96, which was sufficient for genomic diversity analysis as per the guidelines of FAO, 2011) were genotyped on Illumina BovineHD BeadChip (Illumina Inc., San Diego, CA, USA), comprising of 777,962 single nucleotide polymorphisms (SNPs). Various sire families were chosen to encompass a wide range of diversity among the selected animals. The animals included in this research comprised from diverse parities (ranging from 1 to 6 lactations), allowing for the inclusion of lifetime milk yield data. Additionally, animals from different generations (born between 2019 and 2017) were incorporated to account for considerations of adaptability. KF is a crossbred of indigenous T cows crossed with exotic HF, J, and BS, having HF inheritance >50%. PLINK v1.90 [19] software was used for quality control, having the criteria: call rate = 0.90, minor allele frequency = 0.05, and significant departures from Hardy-Weinberg Equilibrium (p-value ≥10^−5^). Sex chromosomes and unmapped SNPs were excluded from this analysis. All experimental procedures involving live animals were approved by the Institutional Animal Ethics Committee (IAEC) of ICAR-NDRI vide Office order 41-IAEC-18-45 dated 27/01/2018.

### Linkage disequilibrium analysis

The D, D′, and r^2^ are the most common estimates for LD. D represents the degree to which two alleles, A and B, are non-randomly linked. It is the difference between the frequency of gametes carrying the pair of alleles A and B at two loci (p_AB_) and the product of the frequencies of those alleles (p_A_ and p_B_). D′ is the ratio of D to its maximum possible absolute value, given the allele frequencies [20].

Since, the value of D is affected by how the alleles are coded (for example, alleles A1 and A2 could be coded as 0 and 1 or 1 and 0), r^2^ is increasingly being utilized to quantify LD [21]. It is also believed to be less susceptible to allele frequency fluctuations and N_e_ changes than other LD measures [20,22, 23]. Further, r^2^ is an LD measure of choice for association studies [24,25]. It is a correlation coefficient of “all or none” indicator variables indicating the presence of A and B which is given as:


$${\text{r}}^{2}={\text{D}}^{2}/{\text{p}}_{\text{A}}(1-{\text{p}}_{\text{A}})\;{\text{p}}_{\text{B}}(1-{\text{p}}_{\text{B}})$$

The r^2^ parameter as the pairwise LD measurements between SNPs was generated using PLINK1.9 [19]. The r^2^ calculation was confined to SNPs within maximum distances of 500 kb because of a very large number of feasible SNP pair-wise comparisons for larger distances. Also, the SNP pairs separated by more than 500 kb tend to show LD values far lower than the useful level of LD r^2^≥0.2 [26].

The decay of the LD was analyzed and plotted by grouping all SNP combinations by their pairwise distance for all the breeds. The LD estimates were obtained for all autosomal SNP pairs binned according to distances of 0 to 10 kb, 10 to 25 kb, 25 to 50 kb, 50 to 100 kb, 100 to 200 kb, and 200 to 500 kb to look at the degree of LD in different breeds.

### Effective population size

Effective population size (N_e_) refers to the size of an idealized population undergoing the same rate of genetic drift as the population being studied [8]. Here we utilized three different methods to obtain comparative estimates of N_e_ in KF cattle.

#### Historical effective population size by SNeP

The pattern of variations in the census population is demonstrated by recent demographic history, as is the variation in the N_e_ of various breeds.

Past N_e_ of all the breeds were derived by SNeP software, utilizing estimates of LD decay in relation to distinct SNP distances. SNeP uses the formula to estimate N_e_ from LD given by Corbin et al [27]:


$${\text{N}}_{T(t)}=\frac{1}{\left(4f(c_{t})\right)}\left(\frac{1}{E[r_{adj}^{2}|c_{t}]}-\alpha \right)$$

Where N_T(t)_ is the effective population size estimated t generations ago in the past c_t_ is the recombination rate t generations ago in the past r^2^_adj_ is the LD estimation, adjusted for sampling bias (r^2^_adj_ = r^2^−(βn)^−1^ where n is the sample size, and β = 1 if the gametic phase is unknown and 2 when it’s known), and α is a correction for the occurrence of mutation (default = 1). Firstly, recombination is inferred between a pair of SNPs considering the relation between physical distance (δ) and linkage distance (d) as *δ* = *kd* (k = 10^−8^). Instead of using the approximation of d = c (1 Mb = 1 cM), the recombination modifier given by Sved and Feldman [28] was used, which is, c = d(1–(d/2)).

The maximum distance between SNPs to be analyzed was kept much higher than the default value to get N_e_ of more recent generations, up to the 5th generation ago (with default settings we were getting N_e_ up to 13 generations ago). The number of bins was also increased to a higher value to get a smoother curve.

#### Recent effective population size by GONE

The GONE software [14] is reported to be reasonably resilient against elements such as population temporal sample heterogeneity, population admixture, genotyping mistakes, and structural split into subpopulations [14].

##### i) Population stratification

We looked for clustering within populations to decide on the appropriate recombination rate to be used later while predicting effective population size by genetic optimization for N_e_ estimation (GONE). A model-based approach by ADMIXTURE [29] was used for grouping individuals from each breed to evaluate the likelihood of the observed data if they’re randomly drawn from a predefined model of the population i.e., K subpopulations. Each population was analysed with tenfold cross-validations from K = 1 to K = 4. The cross-validation procedure allows identifying the value of K for which the model has the best predictive accuracy, as determined by “holding out” data points. Most appropriate K clusters exhibit a low cross-validation error. The ancestry coefficients of the structured population were plotted by the ‘pop-helper’ package [30] in R.

##### ii) Genetic optimization for Ne estimation

Using SNP data from a small sample of current individuals, [14] created an optimization technique called GONE, which employs a genetic algorithm given by Mitchell [31] and works well with a smaller sample size to infer demographic history data. As the physical distance was unknown, we assumed a recombination rate of 1 cM/Mb. Each run comprised 40 replicates. We observed population clustering in HF, so the default recombination rate of 0.05 was adjusted to 0.01 for them. Other settings were used as default in the program. The GONE is an improvement on SNeP and related approaches that employ a relatively oversimplified approach that assumes that LD between loci pairs spanning a genetic distance 1/2t Morgans defines the value of N_e_ at certain generations back in time (t)[14]. These techniques can only show a linear fall in N_e_ and implied demographic history. However, GONE’s methodology was developed on the premise that the value of LD between loci at any given genetic distance results from the cumulative effects of genetic drift and recombination that have been accumulated over previous generations. The N_e_ estimation approach employed by GONE holds a distinct advantage for crossbred cattle populations. It excels in identifying abrupt temporal shifts in population size, which often occur during breed development [14]. These shifts are observable as pronounced declines in the N_e_ trajectory produced by the GONE method.

#### Pedigree-based effective population size

The effective population size (N_e_C_i_) from an increase in coancestry (ΔC_ij_) for all pairs of individuals i and j was estimated according to Cervantes et al [32]. The parameter ΔC_ij_ was computed as


$$\Delta C_{ij}=\sqrt[{\left(t_{i}-t_{j}\right)/2}]{1-C_{ij}}$$

Where *C_ij_* is the coancestry coefficient between the individuals i and j, and *t_i_* and *t_j_* are their equivalent complete generations. Averaging the increase in coancestry for all pairs of individuals in a reference subpopulation, the effective population size based on co-ancestries N_e_C_i_ = 1/2ΔC.

The effective population size based on individual inbreeding rate (N_e_F_i_) [33] was estimated from ΔF, which can be computed by the average in the ΔF_i_ of n individuals.


$$N_{e}F_{i}=\frac{1}{2\Delta F}$$

The number of equivalent subpopulations was computed by Cervantes et al [32]


$$S=\frac{N_{e}C_{i}}{N_{e}F_{i}}$$

## RESULTS

### Quality-checked SNPs used in the investigation

The investigation was carried out on a total of 227 cattle samples, out of which 96 were from KF, 60 from HF, and the rest 71 from the other crossbreds. KF cattle retained the highest proportion of SNPs (86%) after quality checks among all the breeds. Comparatively, other crossbreds also showed higher SNPs passing the quality check thresholds (79% to 86%), compared to purebred HF (74%) (Table 1).

### Linkage disequilibrium analysis

**Figure 1:**
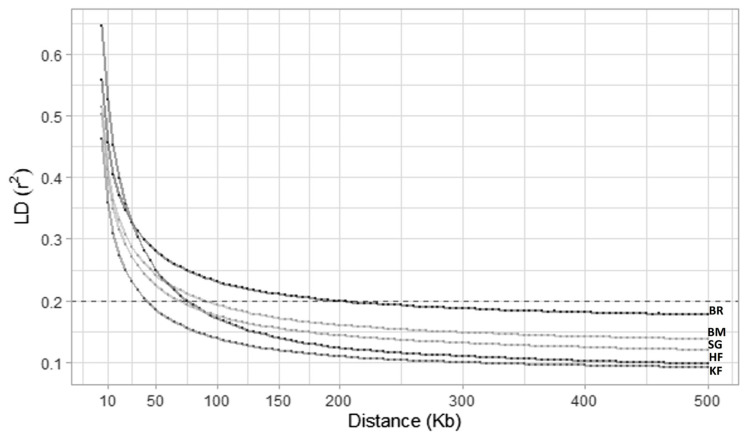
The linkage disequilibrium decay in the explored cattle breeds over the distance of 500 kb. Different rate of linkage disequilibrium decay was observed for Karan Fries (KF), Brangus (BR), Beefmaster (BM), Santa Gertrudis (SG) and Holstein Friesian (HF). The horizontal dashed line is depicting the r^2^ threshold at value of 0.2. KF is showing a steep curvature, indicating rapid decay of linkage disequilibrium. The graph indicates that r^2^ value of 0.2 was achieved at 40 kb inter-marker distance in KF cattle.

On all 29 cattle autosomes, all feasible SNP pairs (on the same chromosome) at less than 500 kb that provided LD values were 93,311,906 (KF), 86,585,073 (SG), and 92,370,033 (BM), 79,099,301 (BR), and 70,826,621 (HF). The mean r^2^ value was the minimum for KF (0.13) and the maximum for BR (0.21). The maximum average r^2^ was observed at a short distance for all studied breeds. The average value of r^2^ steadily declined as the distance between markers increased. HF showed the maximum r^2^ (0.59) at the smallest distance bin (0 to 10 kb), whereas KF manifested the minimum r^2^ (0.10) at the longest distance bin (200 to 500 kb) (Table 2). This was also evident in the LD decay plot (Figure 1), where KF showed a steep curve, decaying rapidly and attaining the lowest position in the plot compared to other studied breeds. Moreover, there were variations in the LD decay rates of the different breeds. LD decay also revealed that to attain an accuracy of 0.85 for genomic breeding values through genomic selection, a necessary level of LD (r^2^) is defined at 0.2 [26]. In the specific context of KF cattle, this r^2^ threshold of 0.2 was reached with an inter-marker distance of 40 kb. To comprehensively cover the autosomal genome (2.51 Gb) of KF cattle for genomic studies, SNPs can be placed equidistantly at 40 kb inter-marker distance (2,510,605 kb/40 kb) which gives a total of 62,675 SNPs.

### Within-breed population stratification

**Figure 2:**
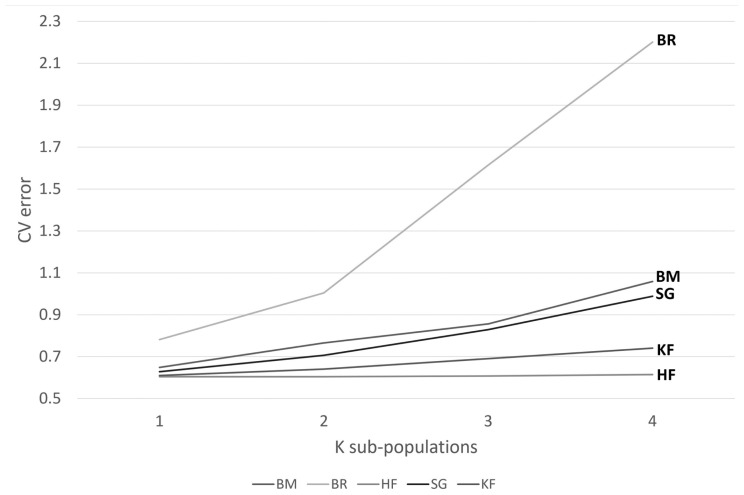
Cross-validation error obtained for each value of K by ADMIXTURE software. Cross-validation (CV) error at different sub-population considerations (1 to 4) for Karan Fries (KF), Brangus (BR), Beefmaster (BM), Santa Gertrudis (SG), and Holstein Friesian (HF). The minimum CV error was obtained at K = 1 for all the crossbreds, whereas for purebred HF it was at 2, indicating structured population.

**Figure 3:**
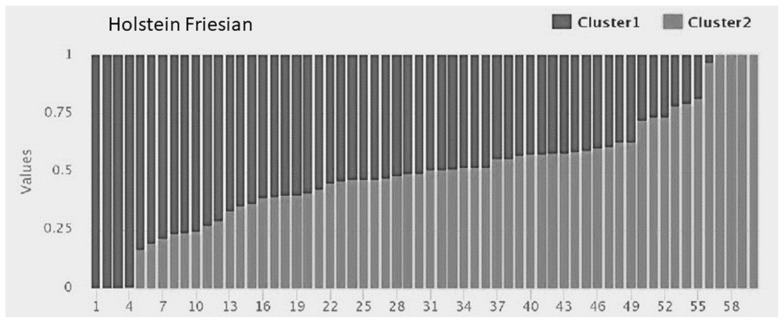
Model-based clustering KF and HF individuals at K = 2 using the program ADMIXTURE. Admixture plot for HF shows structured population which can be seen as two different shades (dark vs light) with individual ancestry coefficients.

The clustering within populations was considered to decide on the appropriate recombination rate to be used later while predicting effective population size. Admixture software showed minimum cross-validation error at K = 1 for all the crossbreds, i.e., KF, SG, BM, and BR, whereas the purebred HF showed clustering at K = 2 (Figure 2). A bar graph demonstrating the ancestry coefficients of HF was shown in Figure 3.

### Effective population size

Effective population size in all the studied breeds was estimated by two different software tools viz. SNeP and GONE. Additionally, pedigree data were also used to estimate the effective population size for KF cattle.

#### Effective population size using SNeP

**Figure 4:**
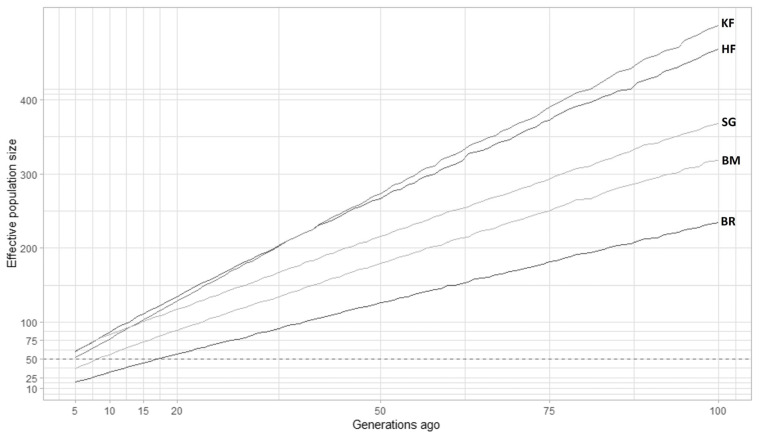
Effective population size estimates (N_e_) by SNeP over 5 to 100 generations ago. The plot depicts the effective population size obtained by SNeP software for Karan Fries (KF), Brangus (BR), Beefmaster (BM), Santa Gertrudis (SG) and Holstein Friesian (HF) over previous 5 to 100 generations ago. The dotted black line represents an N_e_ of 50, which is the UN Food and Agriculture Organization’s threshold for concern. KF, HF, and SG are above this threshold which indicates they have sufficient effective population size.

**Figure 5:**
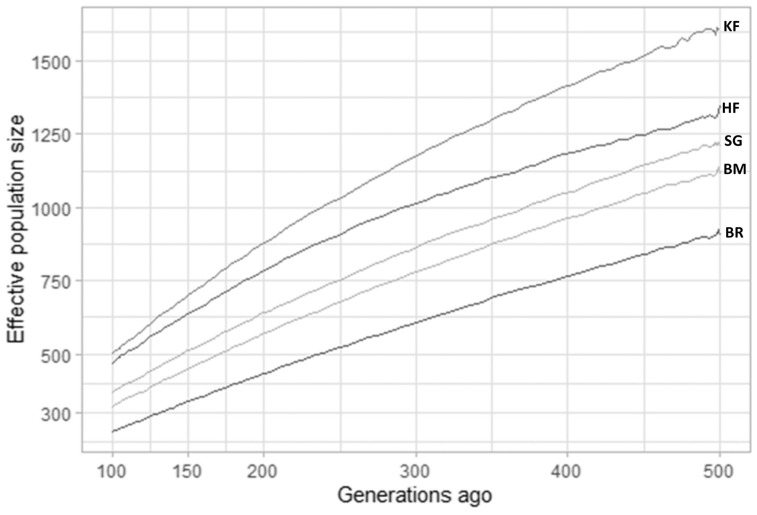
Historical effective population size (N_e_) estimates by SNeP over the previous 500 to 100 generations. Ancestral effective population size estimates previous to 100 generations are illustrated for Karan Fries (KF) Brangus (BR), Beefmaster (BM), Santa Gertrudis (SG) and Holstein Friesian (HF). KF shows highest ancestral effective population size compared to all other crossbreds and HF which indicates it had incorporated maximum diversity.

The LD approach (average r^2^ for markers separated by different genomic regions) was used to estimate N_e_ in the cattle breeds that were investigated. The extent of LD over longer recombination distances was used to estimate recent N_e_, while the extent of LD over shorter distances was used to estimate ancestral N_e_ [34,13]. Figure 4 depicted historical N_e_ (500 to 100 generations ago), whereas Figure 5 depicted more recent N_e_ (100 to 5 generations ago) obtained from SNeP. However, despite all breeds displaying a similar pattern in the curve pattern, the N_e_ values differed across different breeds. The effective population size of all analyzed breeds declined with time, and noticeable variations were observed between breeds. Before 100 generations, N_e_ estimates from SNeP (Figure 4) showed a smooth decline across all the generations for the five observed cattle breeds. This indicated the trajectory of how the historically effective population sizes have shrunk over the past generations. We could observe the rank changes among the breeds in the trajectory. The N_e_ in KF was above all the investigated populations; thereafter it declined below HF around 34 to 40 generations ago and then it further declined below SG in the recent past around 12 generations ago. The estimated values of N_e_ were 52 and 72 at five and 10 generations, respectively in KF cattle using SNeP.

#### Effective population size using GONE

**Figure 6:**
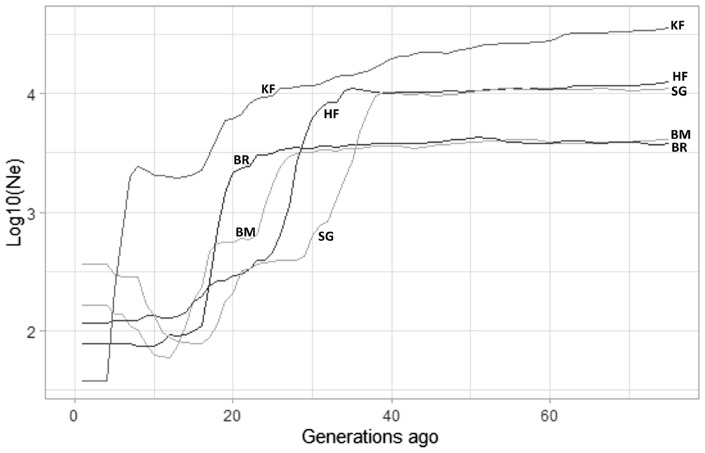
Log10 of effective population size (N_e_) estimates obtained by GONE over the generations. The plot depicts sudden fall in the N_e_ for Karan Fries (KF), Brangus (BR), Beefmaster (BM), Santa Gertrudis (SG) and Holstein Friesian (HF) which corresponds with the time of development of the particular crossbred.

A general non-linear decline was observed in the demographic trajectory, showing changes in the trajectory of the N_e_ of the studied breeds (Supplementary Figure S1). The N_e_ curve pattern plotted from the results of GONE followed a similar trend in all the cattle breeds, where in the past generations the slope is parallel (no change in N_e_), followed by a sharp drop. The N_e_ estimated by GONE 50 generations ago was highest for KF, and its slope declined linearly till 16 generations ago, thereafter it was parallel before the final sudden drop in N_e_. All the breeds recorded a non-linear pattern of the curve. However, there was a significant drop observed in each crossbred cattle (Figure 6). This sudden drop was seen most recently in KF cattle around 5 to 7 generations ago, which could comprehend to the time of its development. The N_e_ values for KF obtained over 5 to 10 generations ago ranged from 219 to 2043, respectively using GONE (Table 3).

#### Pedigree based N_e_

The N_e_ based on increased co-ancestry i.e. N_e_C_i_ (78.56±2.40) was lower in comparison to the individual inbreeding-based N_e_F_i_ (119.15) in KF. The present findings were in accordance with the earlier reports of Santana [35] which indicated the effectiveness of the implemented mating system for controlling inbreeding in the organized herd. The ratio between N_e_C_i_/N_e_F_i_ provided valuable information on population structure [33], since the two parameters are assumed to be measures of the same accumulated drift process, from the foundation of the population to the present time. In the present study, this ratio was 0.65 for KF.

## DISCUSSION

Here we presented the first study on the status of the effective population size (N_e_), demographic trajectory, and LD of the KF cattle using high-density genotype data. The study also included few other crossbreds (BM, SG, BR) and purebred HF cattle, one of the KF’s parental breeds. The investigation revealed a varying degree of effective population size possibly shaped by different demographic events and most importantly, provided information on the breed formation towards the development of this composite cattle.

The observed non-linear pattern of N_e_ curve in cattle breeds could be attributed to a variety of processes that ultimately lead to a decline in the population sizes as a result of genetic drift. N_e_ quantifies the decline in heterozygosity and the percentage rise in inbreeding every generation [36]. The shifts which were observed as sharp drop in the demographic patterns (generation-wise decline in N_e_) could be traced to breed development scenarios. Differential information on N_e_ at various historical periods is provided by LD between pairs of SNPs at various genetic distances. Loosely linked loci indicate population sizes in the recent past, whereas closely linked loci provide estimates of historical population sizes [37]. According to Hayes et al [34], LD between loci with a recombination rate c roughly corresponded to the effective population size of the ancestors 1/(2c) generations ago. This approach on which SNeP relies for estimation of the historical N_e_ is limited to the assumption of linear or steady population. Under this context, recently developed GONE software has been demonstrated with the ability to discern significant shifts in the historical N_e_ [38–42]. A simulation study concluded that the N_e_ derived using GONE reflects genuine demographic changes across generations and is not significantly impacted by selection or the heterogeneity in recombination rate across the genome [39].

If we look at the LD-based N_e_ estimation methods (GONE and SNeP), considering KF development latest in the timeline and comparing the N_e_ of all crossbreds 5 generations ago, the average estimates were 171.38 for GONE and 45.8 for SNeP. KF population at the time of its development was estimated to be 678.5 by GONE, the highest at that time compared to all other breeds. In the field of conservation biology, the “50/500” rule of thumb suggests that the N_e_ of approximately 500 animals is necessary to prevent the loss of genetic diversity due to genetic drift and to maintain a flexible population [43]. Populations with a N_e_ of less than 50 are at risk of extinction without proper management intervention. Therefore, the Food and Agriculture Organization (FAO) has recommended that the minimum level of N_e_ should be at least 50 animals to prevent inbreeding depression. Utilizing three different tools for the estimation of effective population size (N_e_), our study revealed N_e_ values of KF cattle exceeded the FAO recommended level (50); viz. based on pedigree, N_e_ = 78; and LD-based N_e_ estimates using SNeP and GONE were 52 and 219, respectively.

SNeP and GONE, both are LD-based methods but SNeP uses *r*^2^ as an LD estimator while GONE uses *δ*^2^. The SNeP algorithm is based on the relationship between r^2^, N_e_, and c while assuming a linear relationship between N_e_ and the number of generations. GONE returns the geometric mean values of N_e_ over the estimation replicates. When employed on simulated data, GONE performed reasonably resilient against variables such as temporal heterogeneity of population sampling, admixture, subpopulation splits, and genotyping errors [14].

The N_e_ of BM and SG breeds showed an upward trend in recent generations (Table 3), which may be attributed to the introduction of foreign lineages of parental breeds or the crossing of diversified populations. SG was also showing the highest recent N_e_ by SNeP (N_e_ = 61) as well as by GONE (N_e_ = 367). According to previous reports, GONE was better at interpreting recent N_e_ as compared to other coalescence and mutation-recombination-based methods. GONE uses *δ*^2^ to measure LD, instead of r^2^, and there are no analytical remedies for the sampling error of r^2^. Therefore, using it to infer temporal variations of N_e_ poses a challenge. Because of this, it is challenging to forecast with accuracy how drift will affect LD cumulatively over generations, especially when the recombination rate is low [14]. The GONE software has also been reported to work well with a small sample size [38]. When measuring the effective population sizes in turbot, seabream, European seabass, and common carp species of fish using GONE [40] discovered a similar pattern of drastic reduction of N_e_ between the five to nine generations, and these declines might be related to the mixing, founder effect, and artificial selection.

SNeP can effectively determine the ancient demographic history previous to 100 generations [41]. Additionally, SNeP has its default parameter setting based on its estimation procedure at the 13th generation, which is not very recent considering the generation interval of cattle. However, we were able to shrink this up to 5 generations ago by decreasing the minimum distance between SNPs to be analysed. The theory underlying SNeP’s decision to stop estimating in the 13th generation is that the recombination proceeds slowly, having little or no impact on the most recent generations [41].

Similarly, a sharp decline in N_e_ of an ancient, admixed cattle breed-Hawnoo was observed twice in the trajectory, where the first drop (53 to 27 generations ago) was attributed to the advent of selection in cattle breeding, and the second one for the introduction of artificial insemination was 27 to 11 generations ago [44]. This could be the possible reason for the decline observed in N_e_ for HF population estimated by GONE. In the trajectory obtained by GONE, we could observe a drastic decline of N_e_ for all the crossbred cattle (Figure 6; Supplementary Figure S1), which could be the actual origin of these composite cattle groups, as GONE can predict the events of breed formation as well [14]. These phenomena were precisely evident in our study, where BM, BR and SG originated in 1930, 1932, and 1940, respectively [45]. Considering the generation interval of 5 years, they originated 16 to 18 generations ago. A similar drop of N_e_ for KF was found around 5 to 7 generations ago, which corresponded to earlier reports of the development of KF cattle 6 generations ago through genealogical analysis [4]. Martinez et al [42] was also able to determine the time over which different strains of Salmon were established, which corresponded to the most likely generation when the breeding program was started. This information is in concordance with the GONE trajectory getting parallel for these breeds.

Another study concluded that the introduction of new Brahman germplasm from a foreign lineage in the crossbred Braford herd led to a sudden improvement in the declining N_e_ within one generation [46]. In a study on another Indian crossbred cattle Vrindavani, estimates of N_e_ by using SNeP were 53 and 46 at 7 and 5 generations ago, respectively [47]. The chromosome-wise effective population size of Vrindavani cattle was reported by Chhotaray et al [48], where they observed a minimum N_e_ = 22 on Chr 2 and a maximum N_e_ = 38 on Chr 26 and 27 for the recent generations, respectively. While N_e_ estimation by GONE, it was important to look for clustering within the population, which was observed in the HF breed, and accordingly, the value of Haldane correction was considered as 0.01 for the program. Similarly, Fjallnära, Swedish cattle were found to be of admixed origin and showed clustering in the population, was adjusted for its recombination rate accordingly to get better estimates by GONE [41].

The LD between the farthest SNPs determined the N_e_ of the recent most generations [46]. The r^2^ value of 0.2 between the markers can be utilized in genomic studies with at least 80% of accuracy [26], which was achieved at 40 kb inter-marker distance in our population. Our study revealed that if we place a marker equidistantly (at 40 kb interval) within the autosomal genome of 2,510.61 Mb, we can confidently use a custom SNP array of 62,765 markers for genomic studies in KF cattle. This was comparable with the Indian crossbred Vrindavani in which r^2^ value of 0.2 was reported at 25 to 50 kb inter-marker distance, and a similar SNP panel can fit these Indian crossbreds for genomic studies. Similarly, r^2^ value = 0.2 was observed at 40 kb inter marker distance in Hanwoo admixed cattle, and they suggested a comparatively denser panel of 75k SNPs [44].

LD of short inter-marker distance, i.e., 1 to 10 kb was highest for all the crossbreds and has been observed in previous studies as well [46]. In Braford crossbred, the r^2^ value = 0.2 was achieved at 1 to 5 kb inter-marker distance, whereas in its parental breed Hereford it was observed at 40 to 60 kb [46]. The average r^2^ value in KF was 0.41 for 0 to 10 kb inter-marker distance, similar to that of Vrindavani cattle with r^2^ value of 0.46 (Singh et al [47]). These LD estimates were in the range between those reported in taurine cattle (Angus, r^2^ = 0.46; Hereford, r^2^ = 0.49) [49], and indicine cattle (Brahman, r^2^ = 0.25; Nellore, r^2^ = 0.27) [50]. In our study, r^2^ value = 0.59 up to 10 kb distance was found to be the highest in the purebred HF cattle, which was one of the parent breeds of KF.

In conclusion, our study on the comparative assessment of effective population size of KF cattle generated valuable information and provided insight knowledge regarding the population dynamics of this composite cattle. The estimates of effective population size (N_e_) exceeding the minimum recommended level of 50 by the FAO was a desirable characteristic for KF cattle. It may be necessary to improve the effective population size even further in the future to ensure that genetic diversity may not be lost due to random genetic drift. The outcome of the present study assisted in the development of a viable mating plan that uses diverse lines from the population or by introducing a distinct bloodline of parental breeds. Such measures could help to maintain a healthy and resilient population size with a diverse gene pool. The LD decay at 40 kb inter-marker distance indicated that a customized medium-density panel of 63k SNPs would be sufficient to execute genomic selection in the KF population. Our study also suggested possible measures for maintaining appropriate diversity in KF cattle to carry out breed improvement and sustainable utilization programme.

## Figures and Tables

**Table 1:** Sample size and quality check passed single nucleotide polymorphisms for the breeds under study

Breed^1)^	Sample size	QC passed SNPs	

		Number	Proportion
KF	96	670,564	86.20
SG	32	644,326	82.82
BM	23	666,111	85.62
BR	16	615,444	79.10
HF	60	576,441	74.10

SNPs, single nucleotide polymorphisms; QC, quality control.

1) KF, Karan Fries; SG, Santa Gertrudis; BM, Beefmaster; BR, Brangus; HF, Holstein Friesian.

**Table 2:** Summary statistics of linkage disequilibrium r^2^ over increasing distances

Distance	KF^1)^			SG^1)^			BM^1)^			BR^1)^			HF^1)^		
				
	SNP pairs	r^2^	SD	SNP pairs	r^2^	SD	SNP pairs	r^2^	SD	SNP pairs	r^2^	SD	SNP pairs	r^2^	SD
0–10 kb	2,060,374	0.41	0.34	1,937,838	0.45	0.35	2,055,500	0.46	0.35	1,798,607	0.51	0.36	1,759,782	0.59	0.39
10–25 kb	3,050,276	0.28	0.29	2,846,297	0.32	0.31	3,030,881	0.33	0.31	2,620,173	0.37	0.33	2,439,092	0.40	0.36
25–50 kb	4,880,166	0.21	0.24	4,538,892	0.25	0.27	4,838,704	0.26	0.27	4,163,274	0.30	0.30	3,804,291	0.29	0.31
50–100 kb	9,507,427	0.16	0.20	8,831,611	0.19	0.23	9,423,678	0.21	0.24	8,078,226	0.25	0.26	7,286,236	0.20	0.24
100–200 kb	18,686,505	0.12	0.16	17,334,405	0.16	0.19	18,507,554	0.17	0.20	15,829,625	0.21	0.23	14,145,695	0.14	0.18
200–500 kb	55,127,158	0.10	0.13	51,096,030	0.13	0.16	54,513,716	0.15	0.17	46,609,396	0.18	0.21	41,391,525	0.11	0.14
Overall	93,311,906	0.13	0.17	86,585,073	0.16	0.20	92,370,033	0.18	0.21	79,099,301	0.22	0.24	70,826,621	0.15	0.21

SNP, single nucleotide polymorphisms; r^2^, the estimate of linkage disequilibrium; SD, the standard deviation for the r^2^ estimates.

1) KF, Karan Fries; SG, Santa Gertrudis; BM, Beefmaster; BR, Brangus; HF, Holstein Friesian.

**Table 3:** Effective population size (N_e_) values upto 20 generations ago as obtained by GONE

Generations ago	KF^1)^		BM^1)^		BR^1)^		SG^1)^		HF^1)^	
				
	GONE	SNeP	GONE	SNeP	GONE	SNeP	GONE	SNeP	GONE	SNeP
5	219.34	52	139.40	37	77.18	19	298.25	61	122.74	60
6	678.56	57	138.69	41	77.53	21	281.89	66	121.65	65
7	2,001.64	62	111.08	45	77.67	24	286.17	71	120.72	70
8	2,421.11	67	103.45	49	74.31	27	282.73	76	120.65	75
9	2,247.76	72	75.54	53	73.64	29	165.04	80	134.18	81
10	2,042.93	77	61.78	56	73.80	32	133.94	83	135.14	87

N_e_, effective population size; GONE, genetic optimization for Ne estimation.

1) KF, Karan Fries; SG, Santa Gertrudis; BM, Beefmaster; BR, Brangus; HF, Holstein Friesian.

## SUPPLEMENTARY MATERIAL

**Supplementary Figure S1.** Non-linear trend of effective population size estimates by GONE for previous 50 generations.
