# Supplementary material for: Comparative assessment of the effective population size and linkage disequilibrium of Karan Fries cattle revealed viable population dynamics
Source: Anim Biosci. 2023 Nov 2;37(5):795–806. doi: 10.5713/ab.23.0263 (PMC11065711; doi:10.5713/ab.23.0263)
Supplement: Supplementary file 1 [file ab-23-0263-Supplementary-Fig-1.pdf]

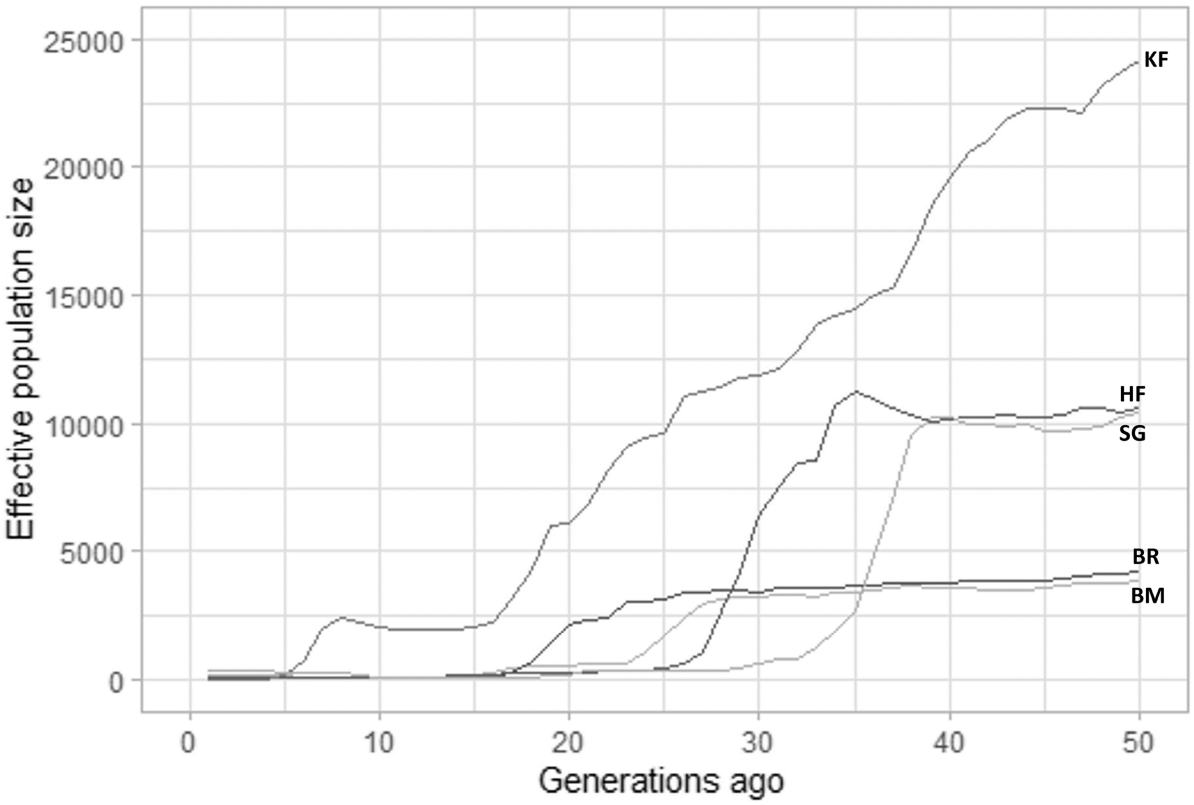

Supplementary Figure S1. Non-linear trend of effective population size estimates by GONE for previous 50 generations. The plot illustrates the effective population size obtained by GONE software for Karan Fries (KF) Brangus (BR), Beefmaster (BM), Santa Gertrudis (SG) and Holstein Friesian (HF).
